# Behind the Smile: Managing Class I Malocclusion and Protrusive Upper Incisors With Kalra Simultaneous Intrusion and Retraction (K-SIR) Mechanics

**DOI:** 10.7759/cureus.65191

**Published:** 2024-07-23

**Authors:** Muskan Chouksey, Kushal Taori, Tikeshwari Gurav, Vikrant Jadhav

**Affiliations:** 1 Orthodontics and Dentofacial Orthopaedics, Sharad Pawar Dental College and Hospital, Datta Meghe Institute of Higher Education and Research, Wardha, IND

**Keywords:** dewey's modification, overbite, overjet, k-sir loop, class i malocclusion

## Abstract

Malocclusion is defined as any deviation from the ideal occlusal positions and the position of the specific teeth. Those that are common are numerous and affect a patient’s stomatology and dental structures in appearance and utility. Class I is the most common type and favors the anterior relationship of both jaws, which lies between the second and third quartiles. Its cause is still unknown at the moment. It is even more frequent than usual occlusion. Class I malocclusion with an overjet of upper incisors less than 4 mm accompanied by type 2 of Dewey’s modification also displays protruded upper incisors and a deep overbite. This case report’s focus is to provide an extensive evaluation of the diagnostic process, management plan, and outcome for a patient who had presented with this specific dental abnormality and simultaneous intrusion and retraction mechanics for an anterior segment with Kalra Simultaneous Intrusion and Retraction (K-SIR) loops.

## Introduction

Orthodontic treatment aims for a proper functional occlusion that is in balance with the surrounding musculature and supporting structures [1]. In addition to compromising the health of oral tissues, malocclusion can result in social and psychological problems [2]. The Angle classification system divides malocclusions into three groups: Class I, Class II, and Class III. The commonest classification of malocclusion is Class I where the buccal groove of the mandibular first molar and the mesio-buccal cusp of the maxillary first molar come into functional contact in the molar area. Despite this strong molar connection, patients with Class I malocclusion may show additional dental anomalies, such as crowding, spacing, rotations, or misalignment of individual teeth [3]. Understanding how and when this malocclusion develops is important to comprehend its etiology. Determining the best course of therapy and aftercare involves a detailed understanding of the etiology and progression of Class I malocclusion. Molar relationships are suitable for Class I patients, however, due to overbite, open bite, rotation, spacing, or even, anterior crossbite their teeth do not lie in the occlusal line, which further in the future leads to occlusal prematurity and temporomandibular joint irregularities [4]. The most common clinical problem is the horizontal shift of the incisors within dental arches; furthermore, there are other dysfunctional occlusal positions in Class I malocclusion, and various other malocclusions. On-demand patients with Class I malocclusion may require orthodontic treatment due to anterior spacing because of its effect on function and esthetics. The term that is used when there are gaps between the anterior teeth and in a Class I skeletal pattern is called Diastema or more specifically Anterior Spacing. This spacing can be due to various reasons like the size of the upper and/or lower jaw and teeth being different, some of the teeth missing, the frenum not correctly attached, one having the habit of pushing the tongue, or inheritance [5].

Similar to the same issue anterior spacing does not always cause a problem in function, but does often cause esthetic concerns that many people wish to address through orthodontic treatment [3]. Anterior spacing affects a patient’s self-esteem mainly when in social-related or even employment-related situations. To this end, it becomes necessary to have a thorough understanding of the causative factor, an accurate diagnostic of the problem, and finally an intervention plan that will cover all aspects of the treatment based on the individual patient’s need. To align the dental arches and to create the proper position of the teeth, it is necessary to utilize braces or other kinds of fixed appliances most often in the case of anterior spacing in Class I occlusion. Facial esthetics, patterns of occlusal connections, and dental, as well as skeletal structure architecture, are aspects that have to be considered all through the planning of the treatments. As discussed earlier, several orthodontic mechanics must be well-coordinated and precisely performed to yield optimal results. However, after all the changes are made, the adjustment stage is followed by the retention stage to ensure they are effective [6]. Loop mechanics in orthodontics is a term used for the wire loops or bends that can be present in the orthodontic wires to cause specific tooth movement. It is possible to apply forces that are added to the task such as rotating, tipping, or aligning the teeth using these loops. Depending on the type of therapy plan and the extent of tooth movement required, specific forms such as omega, T-loops, or Kalra Simultaneous Intrusion and Retraction (K-SIR) or closing types of loops are utilized [7]. It was diagnosed in the same year 1962 that the segmented loop mechanics of Burstone and Nanda had been modified to create the K-SIR arch-wire [8]. It is comprised of a clinically semi-elastic 0.019 × 0.025 inch TMA (titanium-molybdenum alloy) arch-wire and stiffer 7 mm × 2 mm closing loops at the extraction sites [8]. Some authors have pointed out that these loops are evenly located in the flow of the therapy, and the design of the latter explicitly influences the effectiveness of the intervention [9].

In this case, with Class I malocclusion patient with spacing between anterior in the maxillary arch is described along with his diagnosis, course of therapy, and result. The report demonstrates the efficacy of the orthodontic intervention in treating this frequent dental issue by highlighting the clinical and radiographic findings, treatment options used, and the satisfactory resolution of the patient's concerns.

## Case presentation

A 22-year-old male patient arrived at the Out Patient Department of Sharad Pawar Dental College and Hospital, Wardha, and was referred to the Department of Orthodontics and Dentofacial Orthopaedics with the chief complaint of spacing in the upper front region of the jaw along with spacing forwardly placed upper front teeth. There was no relevant medical history or dental history of the patient.

**Figure 1:**
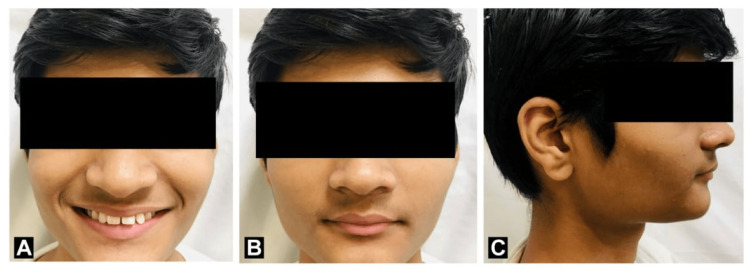
Pre-treatment extraoral photograph. (A) Frontal smiling; (B) Frontal at rest position; (C) Right profile.

In extraoral examination the head form was mesocephalic and the face form was mesoprosopic with an increase in overjet and overbite with acute nasolabial angle and deep mento labial sulcus. The patient's face was symmetrical and his lips were potentially incompetent with a lower lip trap. On temporomandibular joint examination, the temporomandibular joint was normal no signs of clicking, or crepitus were present. The facial profile was convex. On functional examination, it was ruled that spacing in the upper anterior was due to the thumb-sucking habit in childhood as shown in Figure 1.

**Figure 2:**
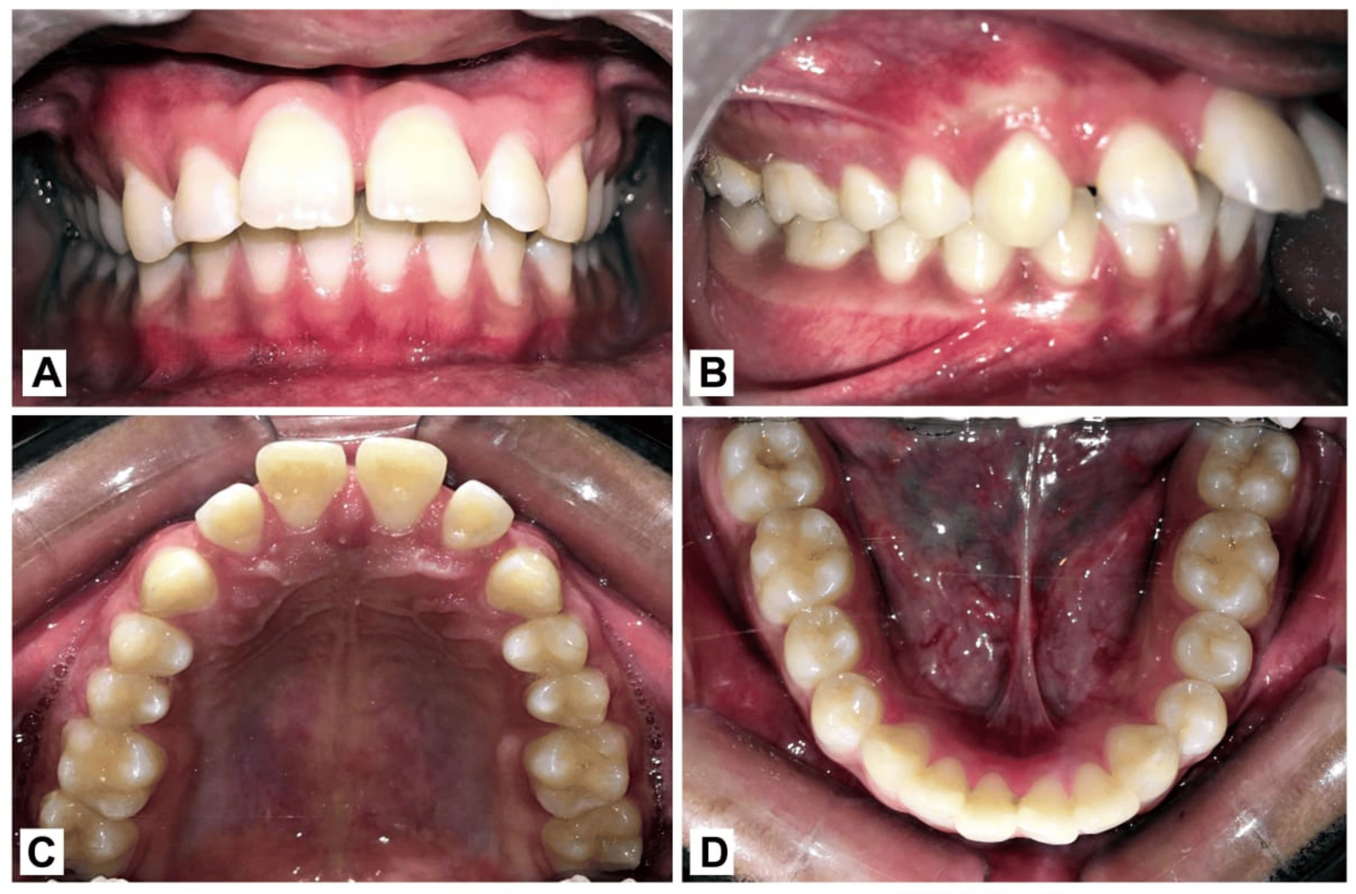
Pre-treatment intraoral photograph. (A) Frontal view; (B) Right buccal occlusal view; (C) Upper occlusal view; (D) Lower occlusal view.

Upon intraoral examination, the posterior buccal occlusion was the proper Class I intercuspation with Class I canine relation on both sides with high labial frenal attachment. The upper anterior showed generalized spacing, whereas the lower arch showed considerable crowding. Each centerline was in line with the others. In both the maxillary and mandibular arches, every incisor, premolar, and molar was present as shown in Figure 2.

**Figure 3:**
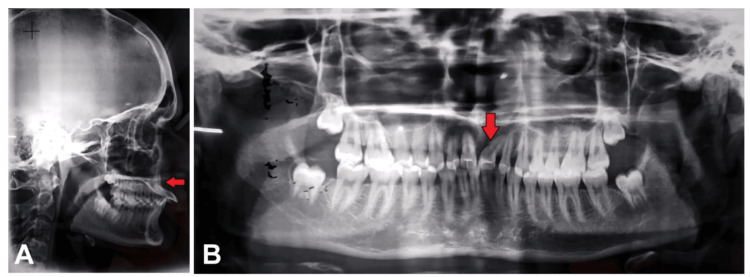
Pre-treatment radiograph. (A) Lateral cephalogram: Appreciate the red arrow, that represents upper incisor roots towards the palatal cortical plate due to severely proclined upper incisors. (B) Orthopantomogram: The red arrow depicts the distal inclination of the root with respect to tooth 21.

On radiographic examination, all the permanent teeth were seen in the orthopantomogram. The cephalometric analysis showed the Class I jaw bases with Dewey’s modification 2 as shown in Figure 3.

Table 1 shows the pre- and post-treatment cephalometric comparison and the difference between them. 

**Table 1:** Lateral cephalogram pre- and post-treatment comparison. SNA: Sella-Nasion-Point A; SNB: Sella-Nasion-Point B; ANB: Point A-Nasion-Point B; FMA: Frankfort-mandibular plane angle; UI: Upper incisor; NA: Nasion-Point A; SN: Sella Nasion; LI : Lower incisor; NB: Nasion-Point B; IMPA: Incisor mandibular plane angle.

Variables	Mean	Pre-treatment	Post-phase II treatment	Difference
SNA (degree)	82±2	84	82	2
SNB (degree)	80±2	81	80	1
ANB (degree)	2±2	3	2	1
Y-axis angle (degree)	53-66	58	57	1
FMA angle (degree)	25	24	24	0
Maxillary dental				
UI to NA (angle)	22	56	24	32
UI to SN (angle)	102±2	140	104	36
Mandibular dental				
LI to NB (angle)	25	27	25	2
IMPA (angle)	90±5	102	93	9
Soft tissue analysis				
Nasomental angle	120-132	114	122	8
Nasolabial angle (degree)	102±4	86	95	9
Upper lip prominence (mm)	1-2	-2 mm	1 mm	3 mm
Lower lip prominence (mm)	1	-2 mm	0 mm	-2
E-line (upper lip/lower lip) (mm)	-4/-2	-4 mm/-3.5 mm	-4 mm/-2 mm	0/1.5
Soft tissue chin thickness (mm)	10-12	9 mm	9 mm	0

Now correlating the clinical and radiologic findings we came to the final diagnosis of Angle Class I malocclusion with Dewey’s modification type 2 (spacing) due to thumb-sucking habit. The treatment objectives were correction of spacing, intrusion of the upper anterior, and simultaneously retraction and intrusion of the upper anterior. Followed by labial frenectomy, soft tissue correction, and relieving crowding in the lower anterior by interproximal stripping.

**Figure 4:**
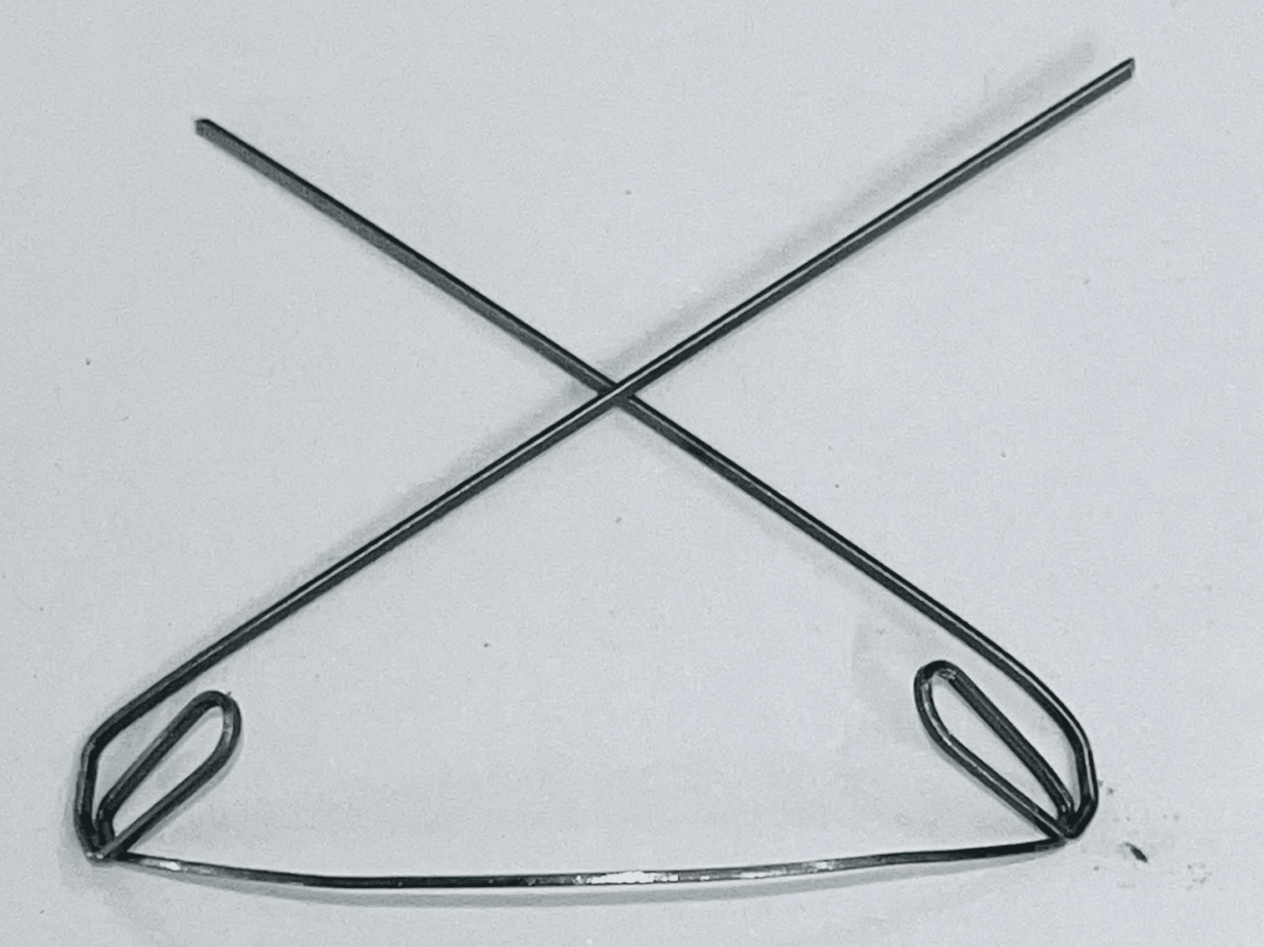
Kalra Simultaneous Intrusion and Retraction (K-SIR) arch. Wire dimension: 0.017 x 0.025 inch; TMA (titanium-molybdenum alloy) loop dimension: 7 mm x 2 mm; α-bend: 45º; β-bend: 45º; pre-activation bends 60º, given 2 mm distal to β-bend; anti-rotation bends 20º.

Then in phase 2, the patient was banded in upper and lower arch metal and Mclaughlin, Bennett, and Trevisi (MBT) brackets of size 0.022 inch slot dimension with the low-profile metal brackets. These Mclaughlin, Bennett, and Trevisi (MBT) brackets work on sliding mechanics with light continuous forces. Then banding was done with all 1st molar for anchorage conservations in the upper arch. The trans palatal arch was given for stability in all three dimensions of space. Initial leveling and alignment of teeth were started which was given by round nickel-titanium wires of size 0.016 inch, 0.018 inch, and 0.016 x 0.022 inch which was then followed by the 0.017 x 0.025 inch nickel-titanium rectangular wires. Further alignment progressed till 0.019 x 0.025 inch nickel-titanium round wires. Simultaneously intrusion and upper anterior retraction were carried through by K-SIR arch-wire of size 0.017 x 0.025 inch using TMA or β titanium wire (TMA) loop as shown in Figure 4 [10].

**Figure 5:**
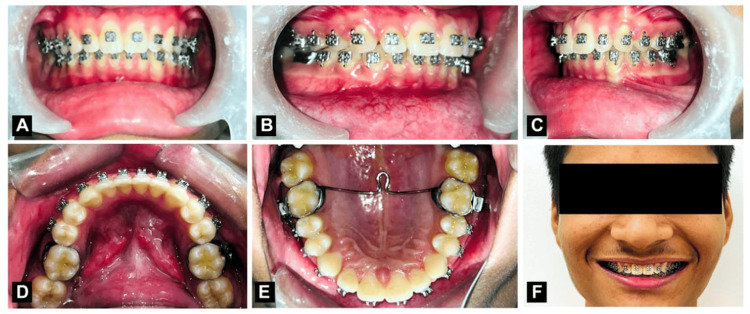
Post-settling photographs. (A) Intraoral frontal view; (B) Right buccal occlusion; (C) Left buccal occlusion; (D) Lower occlusal view; (E) Upper occlusal view with trans palatal arch; (F) Extraoral frontal view.

**Figure 6:**
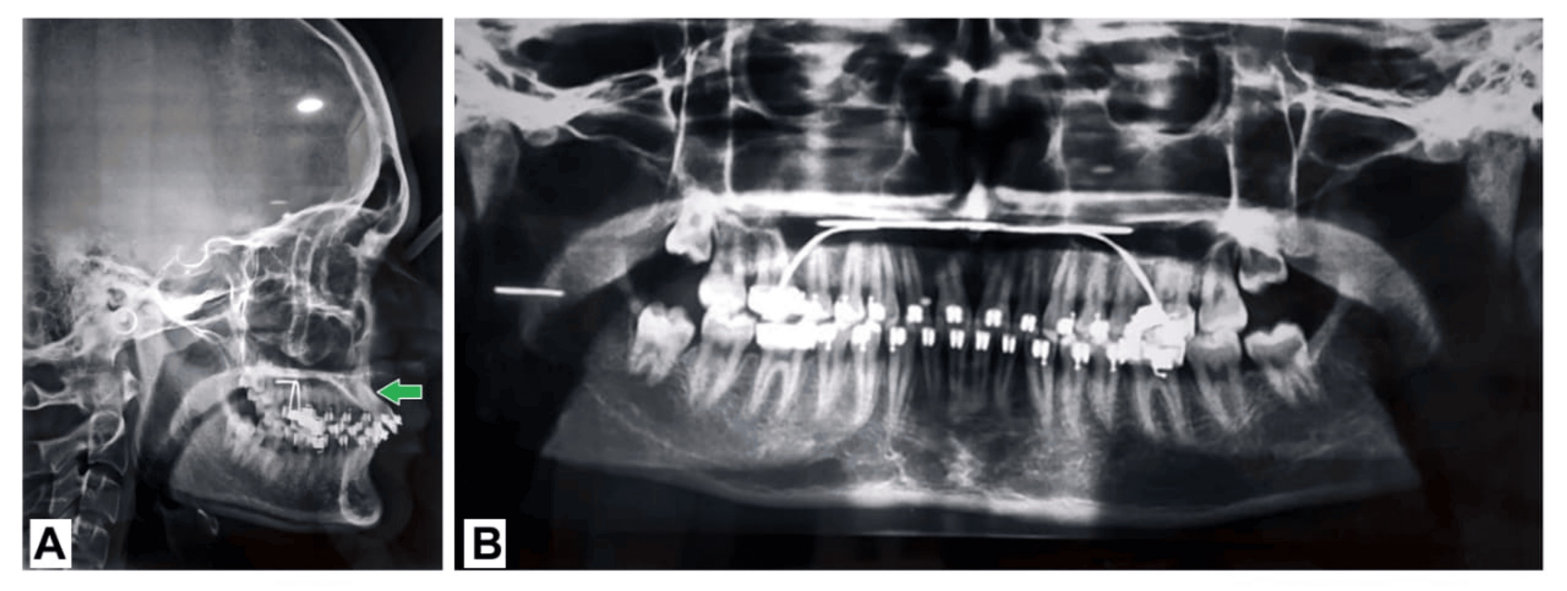
Post-treatment radiographs. (A) Lateral cephalogram: The green arrow depicts that the corrected roots of upper incisors are in cancellous bone (between palatal and buccal cortical plates) normal overjet and overbite achieved. (B) Orthopantomogram: The orthopantomogram depicts corrected crown and root angulations of upper and lower dentition.

The purpose of using these loops was to effect simultaneous intrusion and retraction of upper teeth and reduce the treatment time compared to the edged-wise mechanics with maximum anchorage conservation patients. Lower anterior crowding was reduced by 1.5 mm interproximal reduction using flexible diamond-coated strips to reduce the outer layer of enamel. Then settling of the occlusion was done with the help of elastics given on the crown nickel-titanium 0.018 inch. These elastics help in settling the tooth while swallowing, chewing, biting, and transferring an equal number of forces on the teeth as shown in Figure 5 and Figure 6.

**Figure 7:**
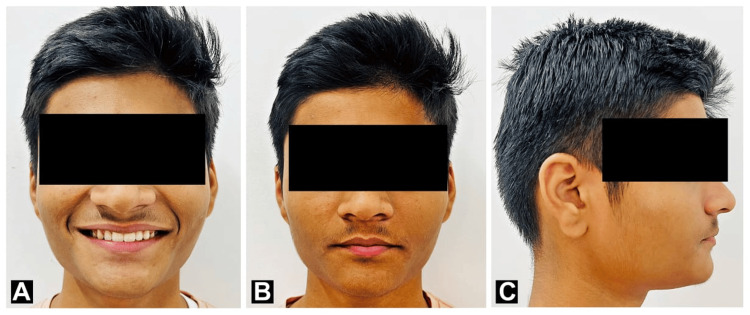
Post-treatment extraoral photograph. (A) Frontal smiling; (B) Frontal at rest position; (C) Right profile.

**Figure 8:**
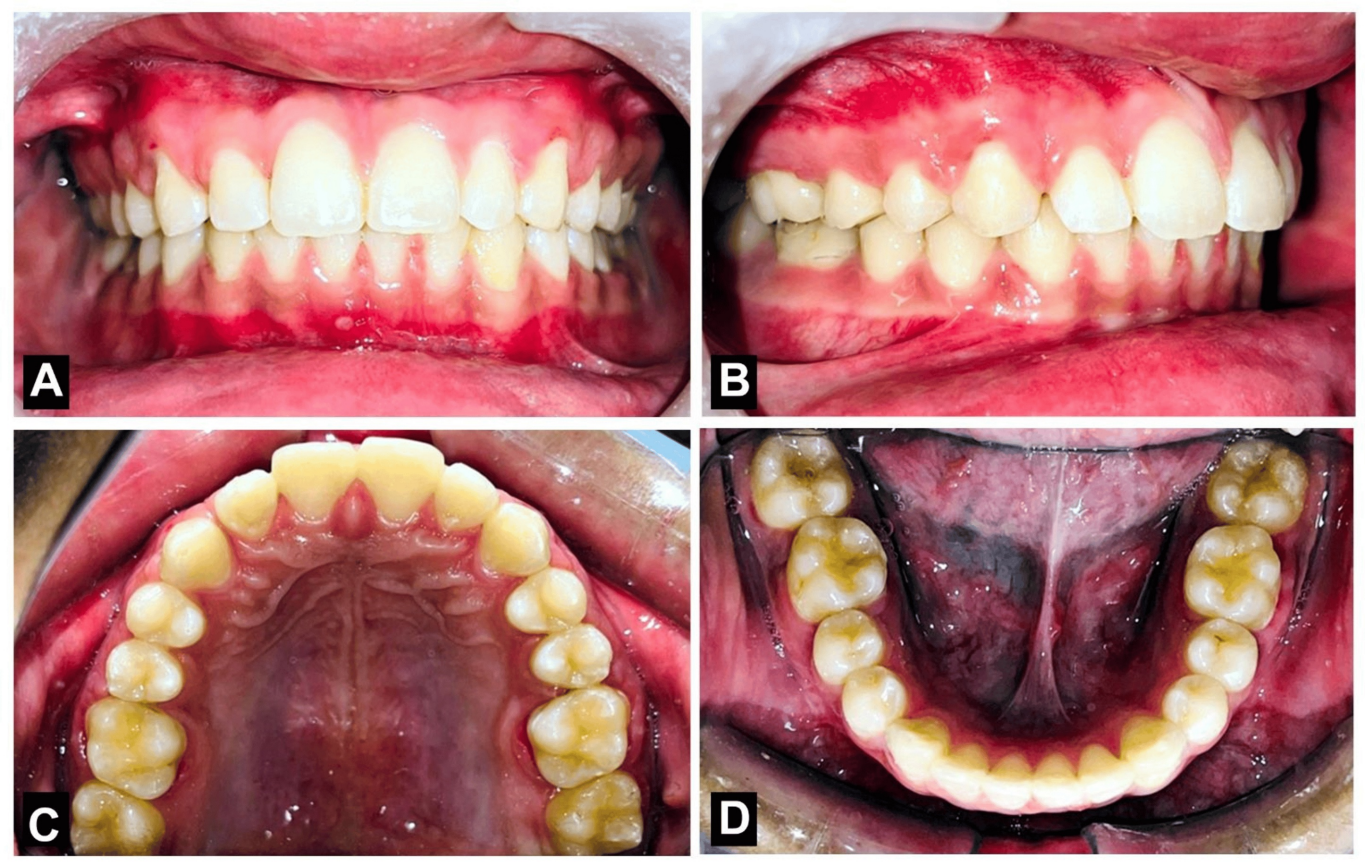
Post-treatment intraoral photograph. (A) Frontal view; (B) Right buccal occlusal view; (C) Upper occlusal view; (D) Lower occlusal view.

Thermoplastic clear retainers, which were placed in the upper and lower arches in combination with a fixed lingual retainer in the upper arch, were used for retention. The purpose of using the retainer was to prevent relapsing of the treatment and maintain the teeth in their new position after the removal of the brackets. The post-treatment outcome was the correction of the acute nasolabial angle, correction of the lip trap, and correction of the profile from convex to straight. correction of the spacing and proclination of the upper anterior teeth by labial frenectomy and using the orthodontic nickel-titanium wires and palatal arch expander, correction of the crowding in the lower anterior by doing interproximal stripping. Thus, the skeletal Class I malocclusion with Dewey’s modification 2 was corrected to near-ideal Class I malocclusion as shown in Figure 7 and Figure 8.

## Discussion

In other words, a proper diagnostic analysis is a crucial element to be made for a treatment plan to be properly set. In this case, cephalometric assessment, radiographic analysis besides clinical examination were used in arriving at the diagnosis. The patient’s x-ray enabled the assessment of the skeletal and dental base while the clinical assessment elucidated the functional interdigitation and dental arch coordination. A cephalometric examination of the patient provided the final diagnosis of Class I malocclusion with anterior spacing in which the normal skeletal connection was ascertained. In such situations, the main goals of the orthodontic treatment include the reduction and elimination of the gaps, achieving the desired level of tooth alignment, and maintaining the continuity of the interdental connections of Class I molars and the canines. The methods involved in the plan of treatment for the patient were applying controlled forces to reposition the teeth correctly and this would require the use of fixed appliances more commonly referred to as braces [11,12]. Therapeutic objectives have always been expected to be attained through orthodontic therapy systematically and scientifically. Maintaining the stability of the occlusion following treatment is the primary goal of orthodontic therapy. To assess the stability of the occlusion, several orthodontic studies have been carried out [13,14]. The type of malocclusion and the patient's compliance are two elements that affect how stable the treatment outcome is, and the growth and motility of the hard and soft tissues. In this case, aesthetics was part of both the diagnosis of the orthodontic problem and the subsequent plan to manage it. Thus, the main goal for this patient was the enhancement of the upper arch spacing inter-arch lower arch crowding concerning the soft tissue. The orthodontic intervention was applied to treat the selected patient who had lower anterior crowding and upper anterior space as an initial condition.

Similar studies by Singh et al. have shown that K-SIR loops are effective in the intrusion retraction and alignment of teeth in Class II malocclusion patients they help reduce the treatment time and help in the patients having maximum anchorage conservation [15]. Similar studies have shown that using light orthodontic forces gets the intrusion along with retraction of anterior teeth in less time compared to the edgewise mechanics along with better stress distribution and controlled tooth movement [16].

## Conclusions

It is crucial to provide a solid basis for the subsequent comprehensive therapy when diagnosing the problem to achieve consistent orthodontic treatment objectives. Thus, we successfully met the need to achieve the occlusion and aesthetic result. The accountability of managing cases as well as constant assessment ensures a desired result within the shortest time possible. The functional occlusion which is to be planned and the maximum intercuspation possible are governed by the size of the teeth present in the upper and lower arches. The final result involved the patient having beautiful teeth they gained well-aligned teeth, upper anterior spacing, and lower crowding were corrected, and overjet and overbite were in the normal range.
